# Intensive Care Unit-Acquired Weakness in Patients With Extracorporeal Membrane Oxygenation Support: Frequency and Clinical Characteristics

**DOI:** 10.3389/fmed.2022.792201

**Published:** 2022-05-10

**Authors:** Xinyi Chen, Xiong Lei, Xin Xu, Yu Zhou, Man Huang

**Affiliations:** Department of General Intensive Care Unit, The Second Affiliated Hospital of Zhejiang University School of Medicine, Hangzhou, China

**Keywords:** ECMO, ICU-acquired weakness, mechanical ventilation, ECMO complications, sedation

## Abstract

**Background:**

Intensive care unit-acquired weakness (ICU-AW) is common in critical illness patients and is well described. Extracorporeal membrane oxygenation (ECMO) is used as a life-saving method and patients with ECMO support often suffer more risk factors of ICU-AW. However, information on the frequency and clinical characteristics of ICU-AW in patients with ECMO support is lacking. Our study aims to clarify the frequency and characteristics of ICU-AW in ECMO patients.

**Methods:**

We conducted a retrospective study, ICU-AW was diagnosed when patients were discharged with a Medical Research Council (MRC) sum score <48. Clinical information was collected from the case report forms. Univariable analysis, LASSO regression analysis, and logistic regression analysis were used to analyze the clinical data of individuals.

**Results:**

In ECMO population, 40 (80%) patients diagnosed with ICU-AW. On univariable analysis, the ICU-AW group had higher Acute Physiology and Chronic Health Evaluation II (APACHE II) [13.9 (6.5–21.3) versus 21.1 (14.3–27.9), *p* = 0.005], longer deep sedation time [2 (0–7) versus 6.5 (3–11), *p* = 0.005], longer mechanical ventilation time [6.8 (2.6–9.3) versus 14.3 (6.6–19.3), *p* = 0.008], lower lowest albumin [26.7 (23.8–29.5) versus 22.1 (18.5–25.7), *p* < 0.001]. The LASSO analysis showed mechanical ventilation time, deep sedation time, deep sedation time during ECMO operation, APACHE II, and lowest albumin level were independent predictors of ICU-AW. To investigate whether ICU-AW occurs more frequently in the ECMO population, we performed a 1:1 matching with patients without ECMO and found there was no difference in the incidence of ICU-AW between the two groups. Logistic regression analysis of combined cohorts showed lowest albumin odds ratio (OR: 1.9, *p* = 0.024), deep sedation time (OR: 1.9, *p* = 0.022), mechanical ventilation time (OR: 2.0, *p* = 0.034), and APACHE II (OR: 2.3, *p* = 0.034) were independent risk factors of ICU-AW, but not ECMO.

**Conclusion:**

The ICU-AW was common with a prevalence of 80% in the ECMO population. Mechanical ventilation time, deep sedation time, deep sedation time during ECMO operation, APACHE II, and lowest albumin level were risk factors of ICU-AW in ECMO population. The ECMO wasn’t an independent risk factor of ICU-AW.

## Introduction

Extracorporeal membrane oxygenation (ECMO) is increasingly being used worldwide in recent years as a rescue therapy for critically ill patients (1). Though ECMO has almost become standard care as a therapy in critically ill patients when less invasive measures have failed, it is potentially associated with serious complications, such as bleeding, infection, acute kidney injury, and neuromuscular complications, which should be considered and weighted (2).

According to the previous studies, intensive care unit-acquired weakness (ICU-AW) can occur in critically ill patients just several days after their ICU admission and muscle loss can exceed 10% during the first week in ICU (3, 4). Muscle strength and mass reduction are common and 43% of critically ill patients have suffered muscle strength decrease (3, 4). It leads to difficulty in weaning off mechanical ventilation, which in turn increases the risk of ICU-AW and diagram dysfunction (5, 6). Moreover, ICU-AW increases the long-term and short-term mortality rates and decreases the life quality when discharged hospital (7).

The ICU-AW is a usual complication in critically ill patients with a prevalence of 43% (interquartile range 25–75%) (4, 8). Patients with ECMO support always suffer more risk factors of ICU-AW such as prolonged mechanical ventilation duration, deep sedation and paralyzed, and long-time of immobility, they are more likely to suffer ICU-AW. Thus, we need to pay more attention to distinguish those who are more vulnerable to muscle weakness and prevent the occurrence of ICU-AW.

Few studies to review the prevalence and clinical characteristics of ICU-AW in patients with ECMO support. The goal of our study is to clarify the clinical characteristics and frequency of ICU-AW in ECMO support individuals. We herein collect clinical information of our patients and analyze their clinical characteristics, hoping to provide some clinical references for our colleagues.

## Materials and Methods

### Study Design and Setting

We conducted a retrospective study of critically ill patients who used ECMO during their ICU stay. Patients were recruited from the general ICU of the second affiliated hospital of Zhejiang University, between March 2017 and March 2020.

### Patients

Patients who had received ECMO therapy during ICU stay were eligible for inclusion criteria in the study. Exclusion criteria were patients who used ECMO to bridge to lung transplantation, experienced cardiac heart arrest, less than 18 years old, had been proven or suspected neurological impairment, using ECMO less than 24 h, had severe head or spinal cord injury and pregnant woman.

we performed a 1:1 matching of each ECMO patient with no ECMO patient who is a statistical twin in terms of APACHE II (intervals of 2 points), SOFA (intervals of 2 points), sex, age group (in 5 years intervals), BMI group, Charlson Comorbidity Index (intervals of 2 points), non-frail before ICU. The 2 of ECMO patients were in the surgical ICU and 48 patients in the medical ICU and 2 surgical ICU patients with ECMO support were matched with 2 surgical patients without ECMO support. A total of 48 ECMO patients in the medical ICU were also matched to corresponding medical ICU patients without ECMO support.

### Limb Muscle Strength

Muscle strength was assessed by physical and occupational therapists on hospital discharge. The strength of muscle of included patients was assessed by Medical Research Council (MRC) score, using a scale from 0 to 5. ICU-AW was diagnosed when a patient is awake and attentive who had a muscle strength sum score <48 out of a maximal score of 60.

### Processes

Following enrollment, demographic data were collected which included gender, age, BMI score, SOFA score, APACHE II, and reasons for admitted to ICU. Therapy details were recorded as well, such as time and depth of sedation (such as deep sedation time and light sedation time), nutrition and glycemic control condition, ventilator settings and duration, drug details, and rehabilitation information. The RASS score was collected from the case reports. The days of light sedation and deep sedation time were collected from the case reports, light sedation was defined as the RASS score between 1 and −2 when using sedative drugs, deep sedation was defined as the deep sedation was defined as RASS score ≤−3 when using sedative drugs (9). The actual calories and protein intake were recorded from the case report form in patients who used enteral nutrition, excepting 1 patient in ICU-AW group used parenteral nutrition. The goal calories were 25–30 kcal/kg and the goal protein intake was 1.2–2.0 g/kg. ECMO settings (such as types of ECMO, rotating speed, gas speed, blood speed, and fraction of inspiration O_2_) and ventilator settings (ventilator mode) were collected 1 day after the ECMO initiation. Complications during ECMO support were also recorded from the case report form and inspection report.

### Statistical Analysis

Continuous variables were expressed as median (median ± interquartile range) or mean (mean standard deviation). Categorical variables were expressed as frequency. Statistical analysis was performed using SPSS, tests exercised were Pearson’s test, Fishers’ exact test, non-parametric test, and logistic regression analysis. Use paired *T*-tests, paired non-parametric tests, and paired Chi-square analysis in the analysis of paired samples. Statistical analyses were performed with SPSS version 23. The R statistical software version 4.1.3 was used to perform the statistical analyses. The LASSO regression analysis was operated with the “glmnet” package. The *p*-value < 0.05 indicated statistical significance.

## Results

**FIGURE 1:**
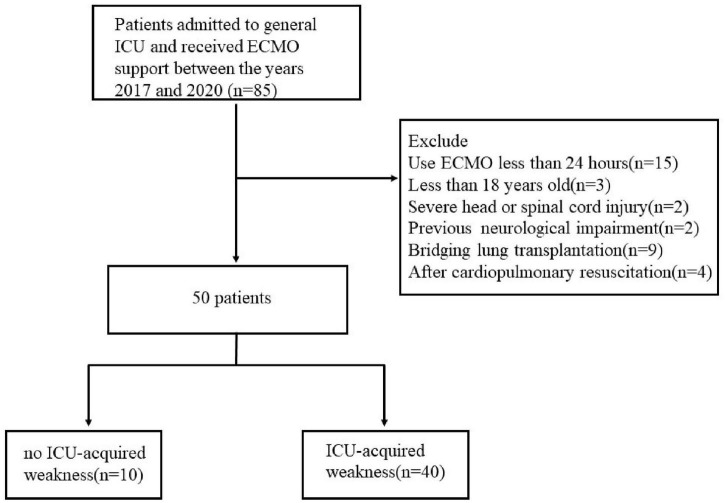
Flow diagram of patients in study.

Over 3 years of the study period, 85 patients who had used ECMO were assessed for eligibility. Among the 85 patients, 35 patients presented exclusion criteria and 50 patients were included in the final analysis (Figure 1). A total of 40 (80%) patients were diagnosed with ICU-AW when discharged.

### Baseline Characteristics of the Study Population

Baseline characteristics of the population were described in Table 1. VV-ECMO and VA-ECMO were most commonly used, there were 22 patients used VV-ECMO support and 27 patients used VA-ECMO support. Severe pneumonia and acute myocardial infarction were most common reasons for the ICU admission. Notably, APACHE II was comparable between 2 groups, patients with ICU-AW had higher APACHE II [13.9 (6.5–21.3) versus 21.1 (14.3–27.9), *p* = 0.005] compared with no ICU-AW.

**TABLE 1:** Baseline characteristics of the study population.

	No ICU-AW (*n* = 10)	ICU-AW (*n* = 40)	*p*-Value
Age, years	44.2 (31–57.4)	55.7 (42.1–69.3)	0.02
Gender, male	8 (16%)	20 (40%)	0.176
Actual weight, kg	75.1 (57.1–93.0)	64 (48.7–79.3)	0.055
Body-mass index, kg/m^2^	25.4 (22.1–29.5)	23.9 (20.2–26.1)	0.235
APACHEII score	13.9 (6.5–21.3)	21.6 (14.3–27.9)	0.005
Diabetes (%)	2 (4%)	4 (8%)	0.744
**Primary diagnosis on admission to intensive care**			
Severe pneumonia (%)	0 (0%)	14 (28%)	
Acute myocardial infarction (%)	3 (6%)	9 (18%)	
Interstitial pneumonia (%)	1 (2%)	5 (10%)	
Acute lung injury (%)	0 (0%)	1 (2%)	
Myocarditis (%)	3 (6%)	2 (4%)	
cardiomyopathy (%)	1 (2%)	2 (4%)	
COPD exacerbation (%)	2 (4%)	0 (0%)	
Pulmonary embolism (%)	0 (0%)	3 (6%)	
Pulmonary hypertension (%)	0 (0%)	1 (2%)	
Pneumothorax (%)	0 (0%)	2 (4%)	
Asthma (%)	0 (0%)	1 (2%)	

### Therapy Details During Intensive Care Unit Stay

Table 2 demonstrated the therapy details of the study population. The deep sedation time during ICU stay differed between 2 groups [2 (0–7) versus 6.5 (3–11), *p* = 0.005]. Meanwhile, the deep sedation time during ECMO support was comparable between 2 groups [1.5 (0–4) versus 4 (3–6), *p* = 0.01]. The ICU-AW group underwent sedation time at a median of 14.5 days whereas the no ICU-AW group underwent a median time of 8 days, but did not differ significantly in 2 groups. The lowest albumin levels differed between 2 groups [26.7 (23.8–29.5) versus 22.1 (18.5–25.7), *p* < 0.001]. However, the average glucose levels and insulin infusion were similar between 2 groups, including the percentage of patients reached goal calories and protein intake.

**TABLE 2:** Therapy details.

	No ICU-AW (*n* = 10)	ICU-AW (*n* = 40)	*p*-Value
**Time and depth of sedation**			
Sedation duration, days	8 (2.8–12.8)	14.5 (6.3–20)	0.078
Deep sedation duration, days	2 (0–7)	6.5 (3–11)	0.005
Light sedation duration, days	4.5 (1.6–9.5)	5 (0.3–12)	0.951
Sedation time during ECMO support, days	5.5 (2.8–6.3)	6 (4–14)	0.151
Deep sedation time during ECMO support, days	1.5 (0–4)	4 (3–6)	0.01
Light sedation time during ECMO support, days	3.5 (0.2–5)	2 (0–6.8)	0.644
Total use of propofol, g	13.25 (4.875–27.5)	14 (4.125–35.25)	0.896
Total use of dexmedetomidine, μg	3200 (1850–5900)	1600 (0–10,600)	0.607
Total use of midazolam, mg	147.5 (0–831.3)	325 (70–1070)	0.369
**Nutrition and glycaemic control**			
Average blood glucose, mmol/L	9.05 (7.71–10.39)	9.5 (8.11–10.91)	0.365
Average fasting blood glucose, mmol/L	8.4 (6.79–10.05)	8.9 (7.26–10.54)	0.405
Patients receiving insulin infusion (%)	4 (8%)	21 (42%)	0.724
Lowest albumin, g/L	26.7 (23.8–29.5)	22.1 (18.5–25.7)	<0.001
Goal calories given during ICU stay (%)	0 (0%)	9 (18%)	0.092
Goal protein given during ICU stay (%)	0 (0%)	8 (16%)	0.184
**Ventilator settings and duration**			
Mechanical ventilation mode 1 day after ECMO initiation			
A/C (VC) (%)	8 (16%)	29 (58%)	
BIPAP (%)	0 (0%)	1 (2%)	
CPAP (%)	0 (0%)	1 (2%)	
IPPV (%)	1 (2%)	7 (14%)	
PCV (%)	0 (0%)	1 (2%)	
PSV (%)	1 (2%)	0 (0%)	
Tracheotomy (%)	0 (0%)	7 (14%)	0.319
Mechanical ventilation time, days	6.8 (2.6–9.3)	14.3 (6.6–19.3)	0.008
Mechanical ventilation time before ECMO initiation, days	0.16 (0.1–0.6)	0.2 (0.1–1.9)	0.409
**Drug details**			
Norepinephrine (%)	10 (20%)	40 (80%)	1
Adrenaline (%)	2 (4%)	23 (46%)	0.077
Sedative drugs (%)	10 (20%)	40 (80%)	1
Neuromuscular blockers (%)	0 (0%)	6 (12%)	0.327
Glucocorticoid (%)	6 (12%)	26 (52%)	1
Glucocorticoid before ICU admission (%)	0 (0%)	4 (8%)	0.696
**Rehabilitation**			
Rehabilitation (%)	2 (4%)	8 (16%)	1
Time to start rehabilitation after entering the ICU, days	4 (2.6–5.4)	10.6 (1.65–19.59)	0.35
Rehabilitation during ICU, days	7.5 (2.55–12.45)	17.87 (6.3–29.44)	0.27
Passive limb movement in bed (%)	2 (4%)	8 (16%)	1
Active body movement in bed (%)	2 (4%)	6 (12%)	0.62
Siting in bed (%)	2 (4%)	4 (8%)	0.33
Standing by the bed (%)	2 (4%)	4 (8%)	0.33
Walking (%)	2 (4%)	1 (2%)	0.067
Limb restraint (%)	10 (20%)	40 (80%)	1
Limb restraint time, days	10.2 (1.4–19)	11.9 (3.42–20.38)	0.576
Highest lactate dehydrogenase level	704 (388.75–827)	2224 (865.75–8320)	<0.001
Highest lactic acid level	3.65 (3–5.65)	15.5 (7.5–22.75)	<0.001

Patients in the ICU-AW group experienced longer ventilation time during ICU stay [6.8 (2.6–9.3) versus 14.3 (6.6–19.3), *p* = 0.008]. Characteristics of mechanical ventilation did not differ significantly between 2 groups, such as the mode, settings of ventilator, and the percentage of tracheotomy. Mechanical ventilation duration before ECMO initiation did not statistically significant between ICU-AW and no ICU-AW group.

In both the ICU-AW and no ICU-AW populations, there were few people participating in rehabilitation, with 2 (4%) patients in no ICU-AW participating in rehabilitation training and 8 (16%) patients in ICU-AW patients participating in rehabilitation training. In the two groups, the time to start rehabilitation and the total time of rehabilitation treatment were not differed significantly, as well as the content of rehabilitation training.

In our analysis, the use of adrenaline during ICU stay was more common in ICU-AW group than in the no ICU-AW group, 26 patients with ICU-AW received adrenaline compared with 2 of patients with no ICU-AW, but they were not statistically significant. Other risk factors such as steroid use and the use of neuromuscular blockade agents did not differ significantly in our analysis. Moreover, there was no difference whether patients participate in rehabilitation exercises.

### Extracorporeal Membrane Oxygenation Management

Extracorporeal membrane oxygenation parameters such as types of ECMO, rotating speed, gas speed, blood speed, fraction of inspiration O_2_, cannulation, and complications during ECMO operation were showed in Table 3. They were not comparable except the occurrence of infection and liver and kidney dysfunction during ECMO support. As compared to the no ICU-AW group, the ICU-AW group had higher percentage of infection during ECMO support [3 (6%) versus 31 (62%), *p* = 0.012] and liver and kidney dysfunction [2 (4%) versus 29 (58%), *p* = 0.007]. The length of ECMO duration appeared no significant difference in our research. Moreover, the ICU-AW group suffers greater financial burden.

**TABLE 3:** Extracorporeal membrane oxygenation management.

	No ICU-AW (*n* = 10)	ICU-AW (*n* = 40)	*p*-Value
**ECMO mode**			
VA ECMO	6 (12%)	21 (42%)	
VV ECMO	4 (8%)	18 (36%)	
VAV ECMO	0 (0%)	1 (2%)	
**ECMO parameters 1 day after ECMO initiation**			
Rotating speed, r/min	3100 (3000–3507.5)	3200 (3000–3500)	0.99
Blood speed, L/min	3.4 (3–4.0)	3.6 (3.2–4.0)	0.52
Gas speed, L/min	3.3 (2.1–4)	4 (3.4–4)	0.242
Fraction of inspiration O_2_ (%)	100 (100–100)	100 (100–100)	0.893
ECMO use, days	5.4 (3.2–7.0)	6.2 (4.3–14.4)	0.207
Lactate dehydrogenase level 1 day before ECMO initiation	335 (250–679.5)	734 (428–1689.5)	0.014
Lactate dehydrogenase level 1 day after ECMO initiation	320 (237–591.5)	748 (444.75–2614.25)	0.003
Lactic acid level 1 day before ECMO initiation	2.1 (1.2–4.2)	2.6 (1.4–11.58)	0.203
Lactic acid level 1 day after ECMO initiation	2.1 (1.2–5.15)	3.5 (1.75–13.1)	0.138
**ECMO cannulation**			
Femoral vein-internal jugular vein (%)	4 (8%)	19 (38%)	
Femoral artery-internal jugular vein (%)	1 (2%)	2 (4%)	
Femoral vein-femoral artery (%)	5 (10%)	19 (38%)	
**Complications of ECMO**			
Bleeding (%)	1 (2%)	5 (10%)	1
Neurological complications (%)	0 (0%)	3 (6%)	1
Liver and kidney dysfunction (%)	2 (4%)	29 (58%)	0.007
Infection (%)	3 (6%)	31 (62%)	0.012
**ICU outcomes**			
ICU duration, days	10.5 (5.9–17.3)	18 (8.8–29.3)	0.19
ICU cost, yuan	188,891 (151,263–239526.3)	412,546 (282190.8–520785.3)	0.004

### LASSO Regression Analysis of Intensive Care Unit-Acquired Weakness

**FIGURE 2:**
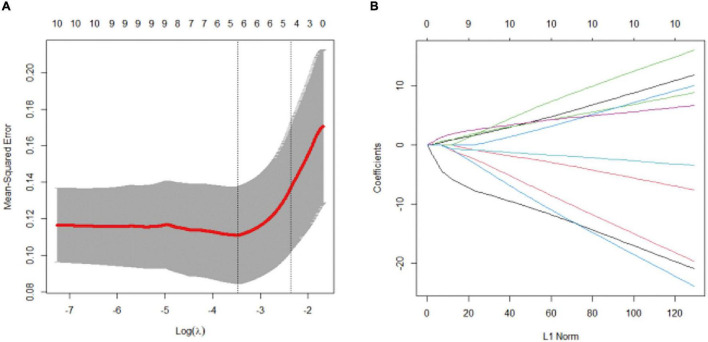
LASSO regression analysis. Variable selection by LASSO binary logistic regression model. **(A)** Selection of tuning parameter (λ) in the LASSO regression using 10-fold cross-validation *via* minimum criteria. five variables with non-zero coefficients were selected by optimal lambda. **(B)** LASSO coefficient profiles for clinical features.

According to the results of the univariate analysis, we selected mechanical ventilation time, mechanical ventilation during ECMO operation, deep sedation time, deep sedation time during ECMO operation, liver and kidney dysfunction, infection during ECMO operation, APACHE II, SOFA, lowest albumin, and ECMO duration into the LASSO regression analysis. The five potential predictors with non-zero coefficients in LASSO analysis were selected, which included the duration of mechanical ventilation, duration of deep sedation, deep sedation time during ECMO operation, APACHE II, and lowest albumin level (Figure 2).

### Comparison of the Occurrence of Intensive Care Unit-Acquired Weakness and Clinical Characteristics Between Patients With Extracorporeal Membrane Oxygenation Support and Without Extracorporeal Membrane Oxygenation Support

To explore whether ICU-AW occurs more frequently in the ECMO population than in the no ECMO population, we performed 1:1 matching of each ECMO patients with no ECMO patients. We found 35 patients in the no ECMO group suffered ICU-AW, 40 patients with ECMO suffered ICU-AW, but there were no significant differences between the two groups. However, we found lower lowest albumin level [23 (19.1–26.9) versus 24.58 (20.8–18.3), *p* = 0.025], higher average blood glucose level [9.7 (8.6–10.5) versus 8.2 (7.2–9.9), *p* = 0.017], longer duration of sedation [11.5 (5.8–19.3) versus 7.5 (4–14.3), *p* = 0.014], especially deep sedation time [6 (3–9.3) versus 3.5 (0.75–5), *p* < 0.001], and more hospitalization costs [363,231 (198,673–477,706) versus 123,768 (67,453–230,473), *p* < 0.001] in ECMO patients (Table 4).

**TABLE 4:** Comparison of the occurrence of ICU-AW and clinical characteristics between patients with ECMO support and without ECMO support.

	ECMO (*n* = 50)	No ECMO (*n* = 50)	*p*-Value
ICU-AW	10 (20%)	15 (30%)	0.123
Age, years	55 (43.3–63.3)	55 (42.5–55.5)	0.442
BMI, kg/m^2^	24.2 (20.8–26.8)	24 (19.6–25.9)	0.105
APACHE II	19 (15–24.3)	19 (15–25)	0.704
SOFA	8.5 (7–11)	9 (7–10.25)	0.825
Charlson comorbidity index	2 (1–3)	2 (1–3)	0.109
Lowest albumin	23 (19.1–26.9)	24.58 (20.8–28.3)	0.025
Average blood glucose, mmol/L	9.7 (8.6–10.5)	8.2 (7.2–9.9)	0.017
ICU duration, days	21.7 (1.9–41.5)	14.4 (4.2–24.6)	0.099
Mechanical ventilation time, days	13.7 (2.9–24.5)	10.4 (1.7–19.1)	0.092
Sedation time, days	11.5 (5.8–19.3)	7.5 (4–14.3)	0.014
Deep sedation time, days	6 (3–9.3)	3.5 (0.75–5)	<0.001
Deep sedation time, days	5 (0.8–11.3)	4 (0–11.3)	0.337
Tracheotomy (%)	7 (14%)	9 (18%)	0.325
Diabetes (%)	6 (12%)	11 (22%)	1
Patients receiving insulin infusion (%)	25 (50%)	15 (30%)	0.355
Glucocorticoid (%)	32 (64%)	21 (42%)	0.352
Neuromuscular blockers (%)	6 (12%)	2 (4%)	1
Norepinephrine (%)	50 (100%)	41 (82%)	1
Adrenaline (%)	25 (50%)	19 (38%)	0.382
ICU cost, yuan	363,231 (198,673–477,706)	123,768 (67,453–230,473)	<0.001

### Logistic Regression Analysis for Predictors of Intensive Care Unit-Acquired Weakness in Combined Cohorts

In the combined cohorts, the clinical characteristics of ICU-AW and no ICU-AW patients were shown in Table 5. In order to further investigate the risk factors of ICU-AW and explore whether ECMO is an independent risk factor of ICU-AW. We performed a LASSO regression analysis and included the use of ECMO, age, APACHE II, SOFA, minimum albumin, average blood glucose, mechanical ventilation time, deep sedation time, sedation time, hospitalization time, and the use of norepinephrine in LASSO regression model (Supplementary Figure 1). The four related variables were selected for the logistic regression analysis, such as APACHE II, minimum albumin, mechanical ventilation time, and deep sedation time. We further performed logistic regression analysis on these four variables and found that they were independent risk factors for the occurrence of ICU-AW. According to logistic regression analysis of combined cohorts, lowest albumin odds ratio (OR: 1.9, *p* = 0.024), deep sedation time (OR: 1.9, *p* = 0.022), mechanical ventilation time (OR: 2.0, *p* = 0.034), and APACHE II (OR: 2.3, *p* = 0.034) were independent risk factors of ICU-AW, but not ECMO (Table 6).

**TABLE 5:** Clinical characteristics of combined cohorts.

	No ICU-AW (*n* = 25)	ICU-AW (*n* = 75)	*p*-Value
ICU-AW	10 (10%)	40 (40%)	0.248
Age, years	47 (34–58)	60 (47–64)	0.006
BMI, kg/m^2^	24.8 (20.5–26.6)	23.5 (20–26)	0.292
APACHE II	15.7 (8.8–22.5)	21 (14–28)	<0.001
SOFA	8 (6–9)	9 (8–11)	0.039
Charlson comorbidity index	2 (1–3)	2 (1–3)	0.62
Lowest albumin	26.5 (23.4–29.6)	22.9 (19.2–26.6)	0.001
Average blood glucose, mmol/L	8.6 (6.9–9.4)	9.42 (7.63–10.54)	0.033
ICU duration, days	10 (5.9–13)	14 (8–26)	0.018
Mechanical ventilation time, days	4 (0.24–8.9)	11.5 (6.6–19)	<0.001
Sedation time, days	0 (0–3.5)	5 (3–9)	<0.001
Deep sedation time, days	3 (0–8)	4 (1–12)	0.193
Deep sedation time, days	4 (1.5–10.5)	11 (6–19)	0.001
Tracheotomy (%)	1 (1%)	15 (15%)	0.115
Diabetes (%)	5 (5%)	12 (12%)	0.645
Patients receiving insulin infusion (%)	7 (7%)	33 (33%)	0.157
Glucocorticoid (%)	11 (11%)	42 (42%)	0.298
Neuromuscular blockers (%)	1 (1%)	8 (8%)	0.545
Norepinephrine (%)	19 (19%)	71 (71%)	0.007
Adrenaline (%)	7 (7%)	37 (37%)	0.063
ICU cost, yuan	103,311 (60,090–200,735)	287,645 (154,936–453,389)	<0.001

**TABLE 6:** Logistic regression analysis of combined cohorts.

Variable	OR	95% CI	*p*-Value
Lowest albumin, g/L	1.9	1.1–3.4	0.024
Deep sedation time during ICU stay, days	1.9	1.1–3.1	0.022
Mechanical ventilation time, days	2.0	1.1–3.7	0.034
APACHE II	2.3	1.1–5.0	0.034

*APACHE II, Acute Physiology and Chronic Health Evaluation II; OR, odds ratio for developing ICU-acquired weakness.*

## Discussion

With the advancements in modern intensive care medicine, mortality of critically ill patients has decreased, however, at the cost of a growing incidence of ICU-AW (10). ICU-AW is known to have detrimental effects on both short-term and long-term clinical outcomes, identifying those who are at high risk of developing ICU-AW is important (7, 11). In our research, we find that the duration of mechanical ventilation, duration of deep sedation, deep sedation time during ECMO operation, APACHE II, and lowest albumin level were independent predictors of ICU-AW in patient with ECMO support.

Our study initially found that ECMO was not an independent risk factor for ICU-AW. But ECMO patients tend to have longer deep sedation time, and lower lowest albumin levels, which were independent risk factors for ICU-AW (5). According to LASSO regression analysis, long deep sedation time, low albumin, prolonged mechanical ventilation time, and high APACHE II were independent predictors of ICU-AW in ECMO patients. Logistic analysis of the combined cohorts also found that APACHE II, minimum albumin, mechanical ventilation time, and deep sedation time are independent risk factors for the occurrence of ICU-AW. These results suggested that deep sedation time, mechanical ventilation time, and albumin levels play a very important role in the occurrence of ICU-AW.

Our analysis shows ICU-AW patients received longer deep sedation time, but not light sedation time. Critically ill patients often receive sedative medications in the ICU, particularly when receiving mechanical ventilation or other invasive intubation, to decrease oxygen consumption, prevent the removal of the tubes and make patients to facilitate treatments and synchrony with the mechanical ventilator (12, 13). However, such deep sedation also makes patients immobile for significantly long periods of time (14). And this kind of deep and continuous sedation with subsequent immobilization can increase the risks of ICU-AW as well as prolonged mechanical ventilation time, which are associated with increased mortality and other negative consequences (9, 15, 16). Work by others finds that daily interruption of sedative infusions can decrease the mechanical ventilation duration and the length of ICU stay (17). Deep sedation in early mechanical ventilation time has been found to be associated with prolonged mechanical ventilation time and increased mortality (9). Therefore, it is of great significance to prevent ICU-AW in critically ill patients and avoid excessive sedation and choose appropriate sedation strategies for different diseases.

In our analysis, we found that ICU-AW patients had a longer mechanical ventilation time compared with no ICU-AW patients and it was independently associated with the occurrence of ICU-AW. Prolonged mechanical ventilation time is an independent risk factor of ICU-AW (11). Because of ventilator−induced diaphragmatic weakness and injury and longtime of immobility, ICU-AW is more likely to happen in patients who received long period of mechanical ventilation, leading to subsequent weaning difficulties and prolonged duration of mechanical ventilation, which can, in turn, increase the mechanical ventilation period and ICU-AW (11). The lengthy periods of ventilatory support contributed to a grip strength decrease in ICU patients, regardless of illness severity (18). ECMO provides important life support in critically ill patients, it improves oxygenation and facilitates protective and ultraprotective mechanical ventilation, minimizing ventilation-induced lung injury (18). Our research showed that the ICU-AW group had longer mechanical ventilation time among patients using ECMO support. Therefore, reducing the duration of mechanical ventilation and early extubation may help to reduce the risk of ICU-AW.

Our analysis shows that ICU-AW patients have lower lowest albumin levels and ICU-AW patients are older than no ICU-AW patients. This indicates that ICU-AW patients have worse nutritional conditions. Other research has also demonstrated that age is associated with the occurrence of ICU-AW (5). We found a higher incidence of ICU-AW in older patients, but it does not show a significant difference in the regression analysis. Early rehabilitation therapy has been shown to be an important mean of reducing the occurrence of ICU-AW, but there are no differences in the ICU-AW and no ICU-AW populations in terms of participation in rehabilitation, initiation and duration of rehabilitation, and patterns of rehabilitation in our experiments (19–22). It might be due to the very small number of people involved in rehabilitation and the fact that we started rehabilitation late in most patients. Other factors such as the use of steroids, the use of neuromuscular blocking agents, glucocorticoids, and rehabilitation didn’t show significantly different in our research, which may partly due to the small number of patients who receive these treatments.

Current treatments of ICU-AW mainly aimed to reduce the risk factors of ICU-AW, such as early rehabilitation, reducing the use of sedative drugs and reduce mechanical duration. However, these treatment methods have limited the outcomes and ICU-AW remains to be a serious clinical problem (5). There were several novel studies showed different methods to which may help to prevent the occurrence of ICU-AW. For example, recently reported clinical research found that obese patients were less likely to have ICU-AW (23). In animal models, ketone diester was found to attenuate skeletal muscle atrophy and inflammation-induced catabolism, which demonstrates the anti-catabolic effects of ketone bodies in muscle atrophy (24). A clinical study also revealed that the ketogenic diet has the potential to improve neurological outcomes for patients with various traumatic injuries (25).

There are some limitations with our research. First, our major limitation is the small number of included patients and this study is based on one center, this might introduce bias and may, therefore, limit the clinical application. Secondly, the dataset is examined retrospectively, which limits the comparison to other studies. Our study primary revealed the frequency and the clinical characteristics of ICU-AW in ECMO support patients. We hope more research to further identify the clinical characteristics and risk factors of ICU-AW in patients with ECMO support, to help in early recognition and treatment of ICU-AW.

## Conclusion

The ICU-AW was common with a prevalence of 80% in ECMO population. Duration of mechanical ventilation, deep sedation time, deep sedation time during ECMO operation, APACHE II, and lowest albumin level were risk factors of ICU-AW in ECMO population. ECMO was not an independent risk factor of ICU-AW.

## Data Availability Statement

The raw data supporting the conclusions of this article will be made available by the authors, without undue reservation.

## Ethics Statement

The studies involving human participants were reviewed and approved by Ethics Committee of the Second Affiliated Hospital of Zhejiang University. Written informed consent from the participants or their legal guardian/next of kin was not required to participate in this study in accordance with the national legislation and the institutional requirements.

## Author Contributions

XC designed the research, analyzed the characteristics of the data, and drafted the manuscript. XL helped in designing the research. XL, XX, and YZ collected the clinical data and helped check the manuscript. MH supervised the research process, provided the clinical reference, and revised the manuscript. All authors read and approved the final manuscript.

## Conflict of Interest

The authors declare that the research was conducted in the absence of any commercial or financial relationships that could be construed as a potential conflict of interest.

## Publisher’s Note

All claims expressed in this article are solely those of the authors and do not necessarily represent those of their affiliated organizations, or those of the publisher, the editors and the reviewers. Any product that may be evaluated in this article, or claim that may be made by its manufacturer, is not guaranteed or endorsed by the publisher.
