# Supplementary figures and images for: Intensive Care Unit-Acquired Weakness in Patients With Extracorporeal Membrane Oxygenation Support: Frequency and Clinical Characteristics
Source: Front Med (Lausanne). 2022 May 10;9:792201. doi: 10.3389/fmed.2022.792201 (PMC9128022; doi:10.3389/fmed.2022.792201)

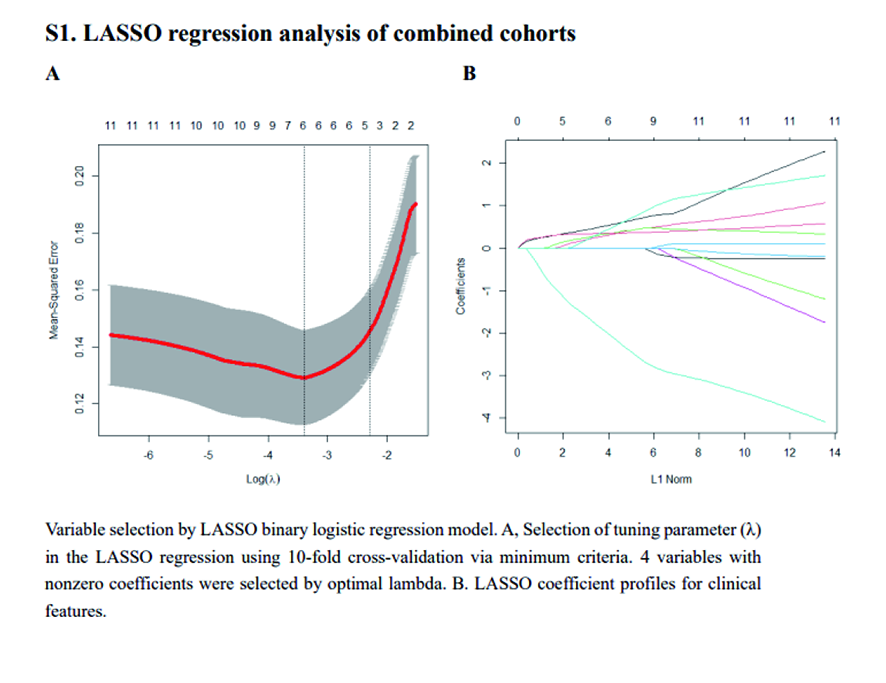

Supplement: Supplementary file 1 [file Image_1.tif]
